# Macrophage depletion attenuates degeneration of spiral ganglion neurons in kanamycin-induced unilateral hearing loss model

**DOI:** 10.1038/s41598-023-43927-9

**Published:** 2023-10-05

**Authors:** Mari Dias Shimada, Masao Noda, Ryota Koshu, Yuji Takaso, Hisashi Sugimoto, Makoto Ito, Tomokazu Yoshizaki, Osamu Hori

**Affiliations:** 1 Department of Otolaryngology, Jichi Medical University, Shimotsuke, Tochigi Japan; 2 Department of Pediatric Otolaryngology, Jichi Children’s Medical Center Tochigi, Jichi Medical University, Shimotsuke, Tochigi Japan; 3 Department of Otolaryngology-Head and Neck Surgery, Graduate School of Medical Sciences, Kanazawa University, Kanazawa, Ishikawa Japan; 4 Department of Neuroanatomy, Graduate School of Medical Sciences, Kanazawa University, Kanazawa, Ishikawa Japan

**Keywords:** Neuroscience, Neurology

## Abstract

Pathological conditions in cochlea, such as ototoxicity, acoustic trauma, and age-related cochlear degeneration, induce cell death in the organ of Corti and degeneration of the spiral ganglion neurons (SGNs). Although macrophages play an essential role after cochlear injury, its role in the SGNs is limitedly understood. We analyzed the status of macrophage activation and neuronal damage in the spiral ganglion after kanamycin-induced unilateral hearing loss in mice. The number of ionized calcium-binding adapter molecule 1 (Iba1)-positive macrophages increased 3 days after unilateral kanamycin injection. Macrophages showed larger cell bodies, suggesting activation status. Interestingly, the number of activating transcription factor 3 (ATF3)-positive-neurons, an indicator of early neuronal damage, also increased at the same timing. In the later stages, the number of macrophages decreased, and the cell bodies became smaller, although the number of neuronal deaths increased. To understand their role in neuronal damage, macrophages were depleted via intraperitoneal injection of clodronate liposome 24 h after kanamycin injection. Macrophage depletion decreased the number of ATF3-positive neurons at day 3 and neuronal death at day 28 in the spiral ganglion following kanamycin injection. Our results suggest that suppression of inflammation by clodronate at early timing can protect spiral ganglion damage following cochlear insult.

## Introduction

An estimated 1.57 billion (95% uncertainty interval 1.51–1.64) individuals reportedly had hearing loss in 2019, accounting for one in five people (20.3% [19.5–21.1]). Among those, about 25.7% (403.3 million people) had hearing loss that was moderate or higher in severity after adjusting for hearing aid use^1^. Hearing loss could occur due to various conditions such as administration of ototoxic reagents including aminoglycoside antibiotics, acoustic trauma, infection, and aging^2^, and interferes with daily communication, which reduces quality of life. Earlier studies have shown that patients with hearing loss experience a higher prevalence of associated adverse health effects than individuals with normal hearing, including social isolation, depression^3,4^, and dementia^5^. Children with hearing loss have reduced educational opportunities including language acquisition and social acquisition^6^ while adults have a much higher unemployment rate compared with those with normal hearing^7^. Even single-sided deafness impairs listening abilities including speech perception in background noise and sound localization^8^.

The pathology of hearing loss includes initial cochlear damage, hair cell death in the organ of Corti, and subsequent nerve degeneration in spiral ganglion^9^. Neuronal cell death in the spiral ganglion prevents restoration of hearing even with cochlear prosthesis. Therefore, preservation of spiral ganglion neurons (SGNs) is crucial for maintaining hearing function during and after pathological situations.

In physiological conditions, the inner ear is protected from inflammatory reaction by the cochlea blood-labyrinth barrier, highly specialized capillary networks in the stria vascularis that selectively pass ions, fluids, and nutrients to the cochlea^10^. Recent studies, however, have revealed that macrophages reside in the different sites of cochlea such as the basilar membrane, osseous spiral lamina, spiral ligament, stria vascularis, and spiral ganglion, and play regulatory roles in the maintenance of cochlear environment^11^. Furthermore, pathological conditions such as insults targeting hair cells induce migration and accumulation of cochlea-resident, and peripheral macrophages especially in specific areas of cochlea as described above^12–14^.

The function of macrophages is generally classified into M1 inflammatory/toxic and M2 anti-inflammatory/tissue repair ones during tissue damage^15^. Although this classification does not meet all pathological conditions, the regulation of macrophage is important to preserve the function in damaged cochlea. For example, in noise-induced hearing loss mouse model, activation of macrophage was observed in cochlea together with induction of IL-1β, a proinflammatory cytokine, 3–7 days after injury, suggesting M1 inflammatory effect in the cochlea^16^. In the same model, macrophage depletion using clodronate liposome suppressed hearing loss. Similarly, blockade of IL-6 with anti-IL-6 receptor antibody improved hearing at 4 kHz in noise-damaged mice cochlea, possibly by suppressing inflammatory responses of cochlear macrophages and preserving SGNs^17^. In an ototoxic injury animal model, expression of fractalkine receptor CX3CR1 on cochlear macrophages has been reported to protect hair cells^14^.

**Figure 1:**
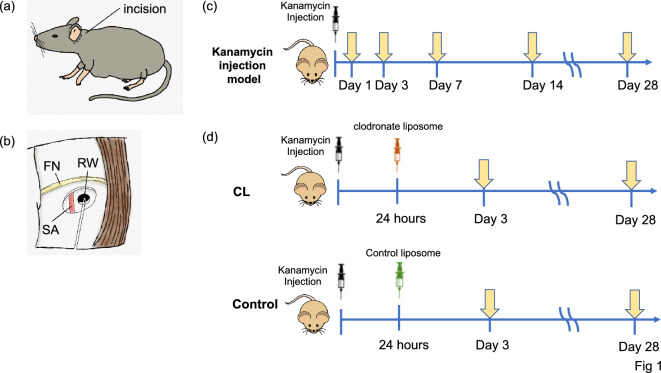
Schematic illustration of the procedures of this study. (**a**, **b**) Schematic illustration of the surgical procedure used for round window injection of kanamycin in mice. RW, round window; FN, facial nerve; SA, stapedial artery. (**c**) Schematic illustration of the experimental schedule for the unilateral hearing loss model. Kanamycin was injected into the left ear of mice through the round window on day 0. Saline was injected into the left ear of mice via same procedure for sham-operated group. Auditory brainstem responses (ABRs) were measured at day 7 and morphological analyses were performed at days 1, 3, 7, 14, and 28. (**d**) Schematic illustration of the experimental schedule for the comparison between clodronate liposome (CL)-treated and control mice. Kanamycin was injected into the left ears at day 0, and CL or control-liposome was administered via intraperitoneal injection 24 h after kanamycin injection. ABRs were measured at day 28 and morphological analyses were performed at days 3 and 28.

Considering the important role of macrophages in the degeneration of SGNs following cochlear damage, in this study, we analyzed the activation status of macrophage and the damage of SGNs using kanamycin-induced unilateral hearing loss model (Fig. 1a–c), in which the direct injection of aminoglycoside caused faster cochlear damage than systemic administration^18^. In addition, we investigated the effect of macrophage depletion on the hearing and degeneration of SGNs by using clodronate-liposome (Fig. 1d).

## Results

### Hearing loss and cochlear damage after unilateral kanamycin injection

**Figure 2:**
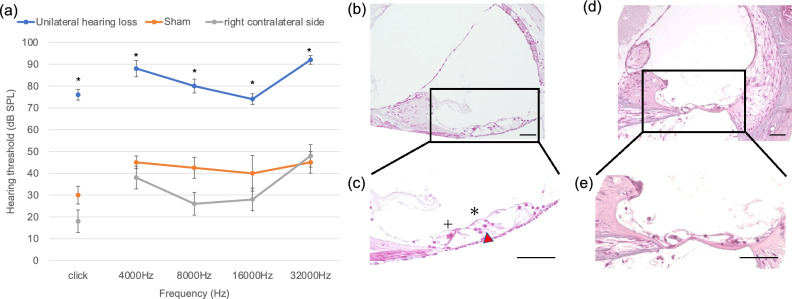
Hearing loss and cell death in the organ of Corti after unilateral kanamycin injection. (**a**) ABRs of kanamycin-injected ears, contralateral ears and sham-operated ears 7 days after operation. Data are expressed as mean ± SEM. The ABR thresholds on the left (deafened) side (blue circles) are elevated for 35–45 dB Sound Pressure Level (SPL) compared with the sham-operated ear (orange circles) across all frequencies. N = 5 each, *p < 0.05 by two-way ANOVA in comparison with sham and right contralateral side. (**b**–**e)** HE stainings to show changes of the cochlear structure following kanamycin-induced unilateral hearing loss. (**b**) Normal structure of the basal turn of the cochlea and **(c)** its magnified image indicating the outer hair cells (*) and inner hair cells (+). Red arrowhead indicates Deiter’s cells. (**d)** Structure of the basal turn of the cochlea 7 days after kanamycin injection and (**e)** its magnified image. In kanamycin-injected ear, loss of inner and outer hair cells, and rupture of reticular lamina and tunnel of Corti are observed, while pillar cells and other supporting cells are partly preserved. Scale bars = 50 μm.

To confirm hearing loss in mice after unilateral kanamycin injection, the auditory brainstem responses (ABRs) were measured at day 7 with a click and four test frequencies (4, 8, 16, and 32 kHz) to the left (ipsilateral, deafened), right (contralateral) and sham-operated (saline-injected) ears. In the deafened side, ABR thresholds were significantly elevated compared with the contralateral side and with sham-operated ears in response to stimuli at every frequency (Fig. 2a), indicating profound unilateral hearing loss after kanamycin injection. These results were consistent with a previous study reporting profound deafness 7 days after kanamycin injection into the left cochlea in mice^19^. Because of the equipmental limitation, we did not apply masking to the contralateral ears. Therefore, there is a possibility that ABR results for kanamycin-treated ears may be worse than presented.

Hematoxylin–Eosin (HE) staining was next performed to analyze the morphological changes of the cochlea 7 days after kanamycin injection. The organ of Corti from kanamycin-injected mice, but not from sham-operated mice, demonstrated loss of outer and inner hair cells, rupture of the reticular lamina and Corti tunnel, but partial preservation of pillar cells and other supporting cells (Fig. 2b–e). Most of the epithelia in the basal turn were severely damaged, while those in the apical and middle turns had tendency to present different level of damages among cochlea (data not shown).

### Degeneration of SGNs after kanamycin injection

**Figure 3:**
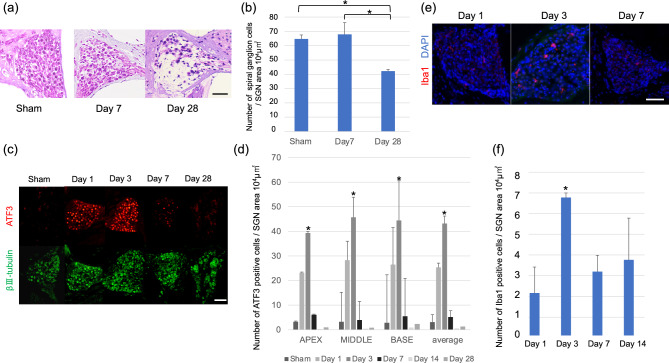
The degeneration of SGNs and activation of macrophages after kanamycin injection. (**a**) HE stainings of spiral ganglion in the basal turn of the cochlea at day 7 after sham-operation, and day 7 or day 28 after kanamycin injection. The SGNs were well preserved at day 7 but were drastically lost at day 28. Scale bar = 50 μm. (**b**) The number of SGNs at day 7 after sham-operation, and day 7 or day 28 after kanamycin injection. N = 4 each, *p < 0.05 by one-way ANOVA followed by Bonferroni post hoc test. (**c**) ATF3 (red) and βIII-tubulin (green) expressions in the SGNs after sham-operated and kanamycin injected conditions. Scale bar = 50 μm. (**d**) The number of ATF3-positive cells in apex, middle, and basal turns, and the average of all turns at day 3 after sham operation and indicated days after kanamycin injection. The number is significantly higher at day 3 after kanamycin injection. N = 4 for day 1, 3, and 14, N = 5 for sham day 3 and day 7 and N = 3 for day 28, *p < 0.05 by one-way ANOVA followed by Bonferroni post hoc test. (**e**) Immunohistochemistry for Iba1 (red) in the spiral ganglion in the basal turn at the indicated days after kanamycin injection. Scale bar = 50 μm. (**f**) The number of Iba1-positive cells in the spiral ganglion at day 1, 3, 7 and 14. N = 4 each, *p < 0.05 by one-way ANOVA followed by Bonferroni post hoc test.

Hematoxylin–Eosin (HE) staining also revealed that the SGNs were well preserved at day 7, when mice presented significant hearing loss, but were only sparsely observed at day 28 after kanamycin injection (Fig. 3a). Quantification confirmed significant reduction of the numbers of SGNs in kanamycin-treated groups at day 28 (Fig. 3b).

To evaluate the damage of SGNs at earlier stages after kanamycin injection, immunohistochemistry for activating transcription factor 3 (ATF3) (red color), a marker of damaged neurons at early stages^20–22^, and βIII-tubulin (green color), a marker of neurons, were performed using sections derived from sham-operated mice and mice 1, 3, 7, 14 and 28 days after kanamycin-induced unilateral hearing loss (Fig. 3c, d). The number of ATF3-positive neurons peaked at day 3, and decreased thereafter in the apex, middle, and basal turns, as well as the average of all turns after kanamycin injection (Fig. 3c, d). In contrast, the number of βIII-tubulin-positive neurons decreased only at day 28 after Kanamycin injection, which was consistent with the results of HE staining (Fig. 3c).

### Microglial activation in the spiral ganglion after kanamycin injection

To assess the activation status of macrophages in the spiral ganglion after kanamycin injection, immunohistochemistry for ionized calcium-binding adapter molecule 1 (Iba1), a highly conserved cytoplasmic protein commonly used as a microglial marker, was performed. The number of Iba1-positive cells increased and peaked 3 days after kanamycin injection. (Fig. 3e, f). Furthermore, Iba1-positive macrophages in the spiral ganglion exhibited larger cell bodies at day 3, suggesting activation of macrophages (Fig. 3e).

### Depletion of macrophage by clodronate liposome (CL)

**Figure 4:**
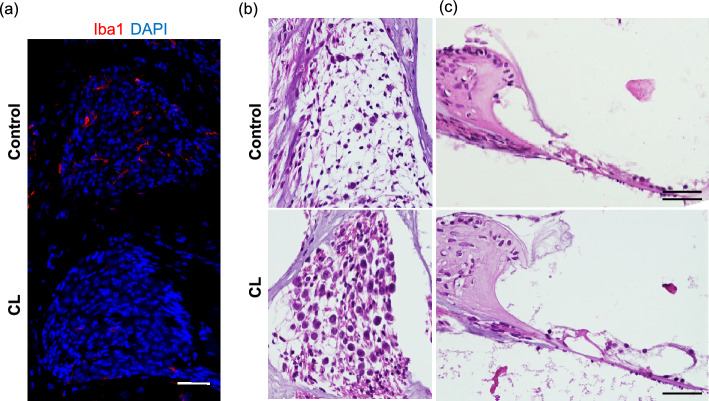
Deletion of macrophages and preservation of SGNs after kanamycin injection. (**a**) Immunohistochemistry for Iba1 (red) and DAPI (blue) staining in the spiral ganglia at day 3 after kanamycin injection at the basal level. The number of Iba1-positive macrophages is much lower in the CL-treated mouse samples; scale bar = 50 μm. (**b**, **c**) HE stainings in the spiral ganglion (**b**) and the organ of Corti (**c**) in control and CL-treated mice at day 28 after kanamycin injection. scale bar = 50 μm. The images are from the basal turn of cochlea.

To investigate the role of macrophages in the SGNs after kanamycin injection, clodronate liposomes (CL) were administered at a dose of 3.6 mg clodronate/mouse 24 h after kanamycin injection. Immunohistochemistry revealed that Iba1-positive macrophages were almost not observed 3 days after kanamycin injection (Fig. 4a). Furthermore, HE staining revealed that SGNs were preserved in CL-treated mice, but not in control liposome-treated mice, at day 28 after kanamycin injection (Fig. 4b). In the organ of Corti, control liposome-treated mice showed severe damages both in hair cells and in other structures such as Corti tunnel and supporting cells. CL-treated mice, in contrast, demonstrated degeneration of hair cells with relatively preserved structures in Corti tunnel and supporting cells (Fig. 4c).

**Figure 5:**
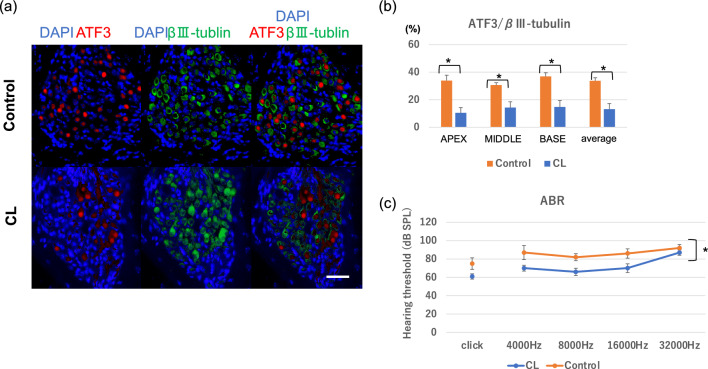
The degeneration of SGNs in control and CL-treated mice after kanamycin injection. (**a**) ATF3 (red) and βIII-tubulin (green) expressions in the SGNs 3 days after kanamycin injection. Scale bar = 50 μm. (**b**) The number of ATF3-positive cells/the number of βIII-tubulin-positive cells in apex, middle, and basal turns, and the average of all turns 3 days after kanamycin injection. The ratio is significantly lower in CL-treated mice. N = 4 each, *p < 0.05 by two-way ANOVA followed by Bonferroni post hoc test. (**c**) Changes of ABRs in control and CL-treated mice 28 days after kanamycin injection. The ABR thresholds in control mice (orange circles) were elevated using 10–15 dB SPL compared with those in CL-treated mice (blue circles) across all frequencies. N = 4 each, *p < 0.05 by two-way ANOVA.

Consistent with the results of HE staining, immunohistochemistry revealed that the CL-treated mice showed significantly less number of ATF3-positive neurons at day 3 after kanamycin injection, suggesting a neuroprotective effect of CL in this model (Fig. 5a, b).

Further ABR analysis revealed a slight threshold shifts in the CL-treated mice, suggesting a partial recovery in the hearing function by CL (Fig. 5c).

## Discussion

In the current study, we first investigated the activation status of macrophages and the damage of SGNs in the course of neurodegeneration after unilateral kanamycin injection. Macrophages in the spiral ganglion enlarged and increased in number at day 3 after kanamycin injection, almost at the same timing as the initial neuronal damage, which was determined by ATF3 expression. Further analysis revealed that pharmacological depletion of macrophages using CL protected SGNs from degeneration, although the hearing function was limited to partial improvement. Our results suggest toxic roles of microglia in the early stage of SGN degeneration after kanamycin injection.

We employed a local injection of kanamycin through the unilateral round window membrane to make a deaf model in mice. Compared with the systemic injection, the local injection of kanamycin can rapidly induce hearing loss and inflammation which includes recruitment of macrophages^23^. Furthermore, this model can minimize the adverse effects of kanamycin including the renal toxicity, and reduce the mortality rate^24^. The protocol is easy and replicable. Bako et.al reported that histological changes of the hair cells and SGNs in locally applied kanamycin and furosemide in guinea pigs^25^. No survival of outer hair cells was observed in the basal and middle turns and the survival rate in the apical turn remained under 10%. Survival of inner hair cells was observed in less than 10% of the basal and apical turns, and no survival was observed in the middle turn. Degeneration of SGNs follows hair cell loss. The averaged densities of SGNs in the deafened ears increased toward the apex. Our results, in contrast, demonstrated no significant differences in the count of SGNs per area among each turn. This discrepancy may be due to the different exposure time to kanamycin, 1–2 h in their model and permanent exposure in our model.

Clodronate (dichloromethylene-bisphosphonate) is a hydrophilic molecule that depletes osteoclasts and has been used as a first-generation bisphosphonate to treat osteoporosis with comparatively fewer side effects than traditional treatments^26^. Previous studies have reported the possibility of using clodronate as a treatment strategy for several conditions. For example, Kato et al.^27^ have reported that clodronate significantly attenuated neuropathic and inflammatory pain unrelated to bone abnormalities via inhibition of vesicular nucleotide transporter, a key molecule for the initiation of purinergic chemical transmission. Clodronate can be encapsulated within phospholipid bilayers that are ingested by macrophages. These liposomes are digested by lysosomal phospholipases, while the clodronate remains in the macrophage. The more phospholipid bilayers and liposomes are ingested by the macrophage, the more clodronate accumulates within the macrophage until it exceeds an intracellular concentration, thereby inducing apoptosis. Indeed, depleting macrophages in the middle cerebral artery occlusion mice model with CL suppressed the activation of microglia in the acute inflammation phase and protected against brain damage^28^.

A previous report demonstrated protection of outer hair cells and preservation of long-term hearing function by CL in a noise-induced hearing loss mouse model^16^. Although it remains unclear why Corti organ morphology and hearing function were only partially restored by CL in our model, we speculate that hair cell damages occurred rapidly after kanamycin injection into cochlea, so that the effect of CL administered 24 h later was limited to degeneration of SCNs which occurred gradually. Therefore, it may be important to perform CL administration in different timings including several hours after kanamycin injection to see the effect to the hair cells.

Regarding the underlying mechanisms, our results suggest a role of neuron-macrophage interaction in the setting of macrophage activation and neurodegeneration in the spiral ganglion after kanamycin administration, as previously reported^29^. Kaur et al., have identified critical roles of Fractalkine (CX_3_CL1)/CX_3_CR1 signaling between neuron and macrophage for the recruitment of macrophage and protection of SGNs from degeneration^23,30^. The discrepancy between their neuroprotective function and our neurotoxic function of macrophage after hair cell damage may suggest the existence of several signaling pathways which determine the protective/toxic fates of macrophage. In this regard, it is noteworthy that ATF3, a transcription factor associated with nerve injury^20–22^, was induced almost at the same timing to microglia activation (Fig. 3c–f). ATF3 can function as a stress-hub gene against different types of stresses such as oxidative stress, cell apoptosis, and endoplasmic reticulum (ER) stress, and it may be a potential therapeutic target for diseases including neurotoxicity in dorsal root ganglion neuron^31^. A recent report demonstrated a cell-type-specific transcriptomic map of the cochlear response to noise, in which ATF3/ATF4 stress-response pathway was robustly induced in the type 1A noise-resilient neurons^32^. It was further reported that aminoglycosides induce misreading in mammalian cells and cause ER stress followed by activation of unfolded protein response (UPR). Intra-tympanic aminoglycoside treatment caused high-frequency hearing loss in XBP1^+/-^ mice and the densities of spiral ganglion cells and synaptic ribbons decreased in that model while sensory cells were preserved. Co-injection of the chemical chaperone tauroursodeoxycholic acid attenuated hearing loss, suggesting that suppressing ER stress in the cochlea could protect the neurons in the spiral ganglion^33^. Further studies are required to clarify the role of ATF3 in the neuron-macrophage interaction and in the neuroprotective/neurotoxic effect of macrophages after cochlear injury.

Besides neuron-macrophage interaction in the spiral ganglion, we speculate that restoration of the organ of Corti by CL may indirectly improve SGN survival. Hayashi et al.^34^ demonstrated that supporting cells in the organ of Corti function as macrophage-like cells, protect adjacent hair cells from pathogens. Similarly, there was a report for the supporting cells to protect hair cells by secreting heat shock protein 70 in ototoxic drug-induced hair cell death model^35^. Therefore, remaining supporting cells may have a beneficial effect to SGNs and macrophages in the spiral ganglion. Further study is required to clarify this point.

In clinical settings, one of the most common and successful surgical approaches for sensorineural deafness is cochlear implantation, which stimulates the auditory nerve directly and transduces sound into neural activity^36^. Following cochlear implantation, the outcomes of hearing function remains variable. Physiological and anatomical variations have been suggested as factors influencing individual differences^37^. The number of remaining spiral ganglion cells is also an important factor^38,39^. Cochlear implant stimulates residual SGNs, hence remaining SGNs may be linked to better surgical outcomes. Our results suggest that regulation of inflammation in the inner ear could preserve SGNs, thereby improving the hearing outcome following cochlear implantation.

In conclusion, we observed activation of macrophage almost at the same timing as the initial neuronal damage in SG after unilateral kanamycin injection, suggesting a role of neuron-macrophage interaction in that model. Furthermore, pharmacological depletion of macrophage using CL protected SGNs against degeneration, suggesting a toxic role of macrophage in the same model.

## Materials and methods

### Experimental animals

Male C57BL6/J mice were obtained from Oriental Yeast Co., Ltd. (Tokyo, Japan) and used in the study at 7–9 weeks of age. Only male mice were used in this study, because there was a report for the sex difference in the rate and severity of sensorineural hearing loss^40^. Mice were socially housed and kept under 12-h dark/light cycle. The relative humidity in the air-conditioned room was maintained at 50–55% and temperature at 21–22 °C. The animals had access to food and water ad libitum throughout the study. All procedures were supervised and approved by the Jichi Medical School Institute of Animal Care and Use Committee (21016-02) and were in accordance with the Japanese Physiological Society’s guidelines for animal care Institutional Review Board and with the ARRIVE guidelines.

### Intracochlear injection of kanamycin

All mice (N = 25) received kanamycin injection through the round window membrane, as previously described^41^. In brief, a small incision was made behind the left ear and the skin was retracted to expose the bulla. A small hole was made in the bulla and 1-μL 30% kanamycin in saline was injected into the cochlea through the round window using a Hamilton syringe. Once kanamycin was injected through the round window, the fascia graft was packed gently around the round window, thus sealing the hole on the round window to prevent the perilymph leaks. Then, the skin overlying the bulla was sutured and the animal was allowed to recover (Fig. 1a, b). Sham-operated group had the same procedure with saline injection to the cochlear instead of kanamycin and was evaluated at post operative day 3 for ATF3 activation and day 7 for degeneration of SGNs.

### Hearing measurement

Animals were randomly divided into five groups and their ABRs were measured at days 1 (N = 5), 3 (N = 5), 7 (N = 5), 14 (N = 5), and 28 (N = 5) to evaluate changes in the hearing threshold after drug administration. ABR recordings and assessments were analyzed by independent researchers blinded to the procedures. The evoked response signal-processing system (Tucker-Davis Technologies and Scope software, Alachua, FL, USA) was used for ABR measurement. Animals were anesthetized with intraperitoneal injections of 0.75 mg/kg medetomidine hydrochloride, 4.0 mg/kg midazolam, and 5.0 mg/kg butorphanol tartrate and then placed in a soundproof chamber. ABRs were administered through tone pips and recorded via subcutaneous electrodes placed in the pinna and vertex, with a ground electrode placed in the back near the tail. Click and tone stimuli of 4, 8, 16, and 32 kHz were generated from 100 to 10 dB in 10 dB steps and finally 5 dB intervals near the threshold; average waveforms were generated from 256 responses. The threshold was determined by a single observer who noted the lowest sound level at which a recognizable waveform was recorded on a screen of tracings stacked from highest to lowest sound level. Waveforms were confirmed as auditory-evoked responses by their increased latency and decreased amplitude as the sound intensity of the stimulus decreased.

### Histology and immunohistochemistry

Mice were anesthetized with an intraperitoneal injection of 0.75 mg/kg medetomidine hydrochloride, 4.0 mg/kg midazolam, and 5.0 mg/kg butorphanol tartrate, then transcardially perfused with 0.01 M phosphate buffered saline (PBS) followed by fixation in 4% paraformaldehyde solution. After decapitation, the temporal bones were dissected and the cochleae were fixed in a 4% paraformaldehyde solution overnight, decalcified with 0.12 M Ethylenediamine tetra acetic acid and cryopreserved in 30% sucrose. The cochleae were rinsed in PBS and dehydrated in 70, 80, and 90% ethanol for 15 min, in 100% ethanol for 30 min and 60 min. Clearing was performed twice with xylol for 30 min. The specimens were subsequently immersed three times in liquid paraffin, blocked using solid paraffin in a paraffin case, and incubated at 46–52 °C for 1 day. The tissues were cut into 4-μm-thick sections and then subjected to HE staining.

For immunohistochemistry, the tissues were cryoprotected in 30% sucrose and sectioned at 10-μm thickness on a horizontal sliding microtome and then processed for immunohistochemistry with ATF3 (Abcam, Cambridge, UK catalog #ab207434), Iba1 (FUJIFILM, Tokyo, Japan catalog #019-19741), and βIII-tubulin (MERCK Millipore, Darmstadt, Germany catalog #AB9354). DAPI (NACALAI TESQUE, INC., Kyoto, Japan catalog #19178-91) was used as nuclear counterstain. Histological observation and image acquisition were conducted using an Olympus BX51 microscope (Olympus, Kyoto, Japan). For quantification, SGNs in basal, middle, and apical turns were discriminated, and the numbers of SGNs were counted per spiral ganglion area using image J software (version 1.50i, Wayne Rasband, National Institutes of Health, Bethesda, MD, USA, https://imagej.nih.gov/ij/). Histological assessments were analyzed by independent researchers blinded to procedures.

### CL injection

Macrophage depletion was induced by CL (N = 16), in which clodronate (18 mg/mL) is encapsulated into liposomes and delivered to the tissues of interests. CL and control (PBS)-encapsulated liposomes were purchased from Hygieia Bioscience (Osaka, Japan) and stored at − 80 °C and until use. We administered 200 μL of CL or control liposome by intraperitoneal injection 24 h after kanamycin injection (N = 8, each). This dose was selected based on the recommendation by the manufacturer and previously published literature^16^. Three days (N = 4, each) and 28 days (N = 4, each) after the surgery, mice were euthanized by perfusion under anesthesia, and the cochleae were collected for immunohistochemical analysis. ABRs were also measured 28 days after kanamycin injection (N = 4, each).

### Statistical analyses

Statistical analyses were conducted using SPSS v. 26.0 software (SPSS Inc. Chicago, IL, USA). The numbers of surviving SGNs, ATF3-positive neurons and Iba1-positive macrophages in the spiral ganglion were evaluated with a one-way analysis of variance (ANOVA). ABR results and comparative analysis of SGNs between the macrophage-depleted and control groups were evaluated with a two-way ANOVA. The Bonferroni post hoc test was used for comparing the conditions. All values are represented as mean ± SEM. p < 0.05 was considered statistically significant.

## Data Availability

Data will be made available upon reasonable request.
